# Classical and specific bioelectrical impedance vector analysis values of children and adolescent athletes according to maturational status: a cross-sectional study

**DOI:** 10.1590/1984-0462/2026/44/2025349

**Published:** 2026-09-07

**Authors:** Leandro Narciso Santiago, Priscila Custódio Martins, Diego Augusto Santos Silva

**Affiliations:** a Universidade Federal de Santa Catarina, Centro de Desportos, Programa de Pós-Graduação em Educação Física, Centro de Pesquisa em Cinantropometria e Desempenho Humano - Florianópolis (SC), Brazil.; b Universidade do Extremo Sul Catarinense, Curso de Educação Física - Criciúma (SC), Brazil.

**Keywords:** Athletes, Bioelectrical impedance, Body composition, Sexual maturation, Adolescents, Atletas, Bioimpedância elétrica, Composição corporal, Maturidade sexual, Adolescentes

## Abstract

**Objective:**

The aim of this study was to analyze classic (BIVAc) and specific (BIVAsp) bioelectrical impedance vector analysis values of children and adolescent athletes according to their maturational status.

**Methods:**

A total of 78 subjects (36 male and 42 female), classified as prepubertal and pubertal, participated in the study. Bioelectrical parameters such as resistance (R), reactance (Xc), and impedance (Z) were measured using a tetrapolar bioelectrical impedance analysis (BIA) device (Sanny^®^ BIA1011AF, 50 kHz, 800 μA) and subsequently normalized by height (BIVAc) and by body circumferences and height (BIVAsp). Five adjustment models were developed using analysis of covariance: raw; sex; sex and age; sex, age, and training experience; and sex, age, training experience, and weekly training volume.

**Results:**

Prepubertal children and adolescent athletes displayed higher raw bioelectrical values (R, Xc, and Z) than pubertal athletes, even after adjusting for covariates. The BIVAc approach yielded higher values across all adjustment models (p<0.01). In contrast, no significant differences were observed between prepubertal and pubertal groups in the BIVAsp approach, even after adjusting for all models (p>0.05).

**Conclusions:**

Prepubertal children and adolescent athletes exhibit higher values of R, Xc, and Z than pubertal athletes, both in raw analyses and when normalized for height (BIVAc). However, when normalized by body circumferences and height (BIVAsp), these differences were no longer significant, suggesting that height normalization preserves maturational differences, whereas normalization by body circumferences attenuates them.

## RESUMO

**Objetivo:**

Analisar os valores de análise vetorial de impedância bioelétrica clássica (BIVAc) e específica (BIVAsp) em crianças e adolescentes atletas, de acordo com seu estado maturacional.

**Métodos:**

Participaram do estudo 78 indivíduos (36 do sexo masculino e 42 do feminino), classificados como pré-púberes e púberes. Parâmetros bioelétricos como resistência (R), reatância (Xc) e impedância (Z) foram coletados usando um dispositivo de BIA tetrapolar (Sanny^®^ BIA1011AF, 50 kHz, 800 μA) e posteriormente normalizados pela estatura (BIVAc) e pelas circunferências corporais e estatura (BIVAsp). Cinco modelos de ajuste foram desenvolvidos por meio de análise de covariância (ANCOVA): bruto; sexo; sexo e idade; sexo, idade e experiência de treino; e sexo, idade, experiência de treino e volume semanal de treino.

**Resultados:**

Crianças e adolescentes pré-púberes apresentaram valores bioelétricos brutos (R, Xc e Z) mais elevados do que os púberes, mesmo após ajuste para todas as covariáveis. A abordagem BIVAc resultou em valores mais altos em todos os modelos de ajuste (p<0.01). Em contraste, não foram observadas diferenças significativas entre os grupos pré-púbere e púbere na abordagem BIVAsp, mesmo após o ajuste para todos os modelos (p>0.05).

**Conclusões:**

Crianças e adolescentes atletas pré-púberes apresentam valores mais elevados de R, Xc e Z em comparação aos púberes, tanto nas análises brutas quanto nas normalizadas pela estatura (BIVAc). Entretanto, quando normalizadas pelas circunferências corporais e estatura (BIVAsp), essas diferenças deixam de ser significativas, sugerindo que a normalização pela estatura preserva diferenças maturacionais, enquanto a normalização pelos perímetros corporais as atenua.

## INTRODUCTION

Body composition assessment is a key tool for monitoring and optimizing athlete performance, particularly during growth phases such as puberty. During this stage, adolescents undergo substantial changes in muscle mass, fat distribution, and hydration status, directly influencing physical capacity and performance.^1^ ^,^ ^2^ Assessing body composition enables the adjustment of nutritional and training strategies, supporting performance, metabolic efficiency, and injury prevention.^3^

In the sports context, evaluating muscle mass, body fat, and body water provides essential information to guide interventions to improve performance and reduce injury risk. Muscle mass is closely related to strength and power, while excess body fat may impair performance. Adequate hydration is also critical for maintaining electrolyte balance and recovery.^3^ ^,^ ^4^

Bioelectrical impedance analysis (BIA) is widely used due to its practicality and rapid assessment of body composition.^5^ However, its interpretation can be influenced by predictive equations and maturational status, potentially introducing bias.^6^ Therefore, the use of raw bioelectrical parameters - resistance (R), reactance (Xc), and impedance (Z) - has been proposed to improve analytical consistency.

In this context, bioelectrical impedance vector analysis (BIVA) provides a qualitative interpretation of tissue electrical properties using raw parameters.^7^ Two approaches are commonly used: classical BIVA (BIVAc), which normalizes parameters by height, and specific BIVA (BIVAsp), which additionally accounts for body circumferences (arm, waist, and calf) to adjust for body geometry.^8^ ^,^ ^9^

A recent systematic review demonstrated that both BIVAc and BIVAsp have been applied in pediatric populations, including young athletes, although evidence comparing these approaches across maturational stages remains limited.^10^ In particular, few studies have examined how different normalization strategies behave in relation to pubertal changes in body composition. Therefore, the objective of this study was to analyze the values of BIVAc and BIVAsp in children and adolescent athletes according to maturational status.

## METHOD

This cross-sectional study is part of the project “Health Conditions of Athletic and Non-Athletic Children and Adolescents,” developed at the Federal University of Santa Catarina (UFSC). Data were collected in Florianópolis and Itapema, Santa Catarina, Brazil, between September 2023 and April 2024. Bioelectrical data were collected at UFSC, while anthropometric and maturational assessments were conducted at the athletes’ training facilities.

The study was approved by the UFSC Human Research Ethics Committee (protocol 69422423.4.0000.0121; approval 6.107.556) and adheres to the STROBE statement. Participants provided assent, and parents or guardians signed informed consent forms.

A convenience sample of athletes aged 6-18 years who participated in rhythmic gymnastics, surfing, skateboarding, futsal, soccer, and volleyball was included. All eligible athletes were invited to preserve the ecological characteristics of the sample. An athlete was defined as an individual engaged in regular training and participation in competitive events at local, state, national, or international levels.

The inclusion criteria were:

1. Athletes aged 6-18 years competing at local to international levels; and
2. Residence in Itapema, Santa Catarina, Brazil.

The exclusion criteria were:

1. Use of controlled medication;
2. Orthopedic injury at assessment;
3. Physical disability.

Because this study used a convenience sample derived from a larger project, the final sample size was determined by participant availability rather than an a priori calculation. Therefore, a post-hoc power analysis was conducted using G*Power^®^ (version 3.1.9.7; Universität Düsseldorf, Germany), adopting the F-test family and the analysis of covariance (ANCOVA) model (fixed effects, main effects, and interactions), consistent with the analytical approach of the study. Parameters were set at α=0.05, and power (1−β)=0.80.^11^ The sample size (n=34) was sufficient to detect medium effect sizes (f=0.50).

BIA was assessed using a tetrapolar device (Sanny^®^, Brazil; 50 kHz, 800 μA), providing raw values of resistance (R), reactance (Xc), and impedance (Z). Participants followed standard pre-assessment procedures, including fasting (≥4 h), avoidance of caffeine (12 h), alcohol (48 h), and intense physical activity (24 h).^12^ On the day of assessment, the participants were instructed to wear light clothing, remove all metallic objects (e.g., rings, earrings), and remain in the supine position for 10 minutes before measurement.^12^ Height was measured using a stadiometer, while arm, waist, and calf circumferences were obtained using a non-elastic tape.

The dependent variables were derived from BIVAc and BIVAsp. For BIVAc, resistance and reactance were normalized by height (R/H; Xc/H) and plotted on an RXc graph with 50, 75, and 95% tolerance ellipses based on a reference population of children and adolescents.^13^ ^,^ ^14^ Vector position and displacement were interpreted as indicators of tissue electrical properties, with the major axis reflecting hydration status and the minor axis reflecting body cell mass.^8^ For BIVAsp, resistance and reactance were adjusted using a geometric correction factor (area/length) derived from height and body circumferences (arm, waist, and calf), generating specific resistance (Rsp) and reactance (Xcsp). Specific impedance (Zsp) was calculated as (Rsp^2^+Xcsp^2^)^0.5^.^8^ All BIVAsp variables were treated as continuous and expressed in ohms (Ω).

The independent variable, sexual maturation, was assessed following the criteria proposed by Tanner to determine the adolescent’s maturational stage,^15^ which has been validated in the Brazilian population.^16^ For both sexes, illustrations of pubic hair development were used to determine maturational status. Adolescents were categorized as prepubertal (stage 1), pubertal (stages 2, 3, and 4), and postpubertal (stage 5). Self-assessment of pubic hair development has been shown to be more effective than self-assessment of genital development in male adolescents and more effective than self-assessment of breast development in female children and adolescents.^17^ ^,^ ^18^

The control variables were sex, age, years of sports practice, and weekly training volume. Sex was also used as a stratification variable (male and female) due to known differences in bioelectrical parameters, with males typically exhibiting lower R and Xc values than females, largely attributable to higher muscle mass and lower adipose tissue.^19^ ^,^ ^20^

Age was collected in complete years and treated as a continuous variable, given its association with changes in bioelectrical parameters across growth and development.^19^ Years of sports practice were recorded in full years, as longer training exposure is associated with improvements in body composition and bioelectrical characteristics.^6^ Weekly training volume was measured in hours per week, considering that cumulative training load may influence physiological adaptations and body composition in adolescent athletes.^21^ All control variables were obtained through a structured questionnaire completed by the participants or, for those under 12 years of age, by their parents or guardians.

Pubertal and post-pubertal participants were grouped into a single category (pubertal) due to the small number of post-pubertal individuals (n=5), aiming to increase statistical power. This approach is supported by the similar physiological and developmental characteristics shared between these stages, particularly in athletic populations.^22^

Data distribution was assessed using the Shapiro-Wilk test. Variables with normal distribution are presented as mean±SD, while non-normally distributed variables are presented as median. Between-group comparisons were performed using Student’s t-test or Mann-Whitney U test, as appropriate. Effect size was calculated using Cohen’s d.^23^ To examine differences in bioelectrical parameters (raw, BIVAc, and BIVAsp) between prepubertal and pubertal groups, ANCOVA was applied using five models: Model 1 (unadjusted); Model 2 (adjusted for sex); Model 3 (sex and age); Model 4 (sex, age, and years of sports practice); and Model 5 (sex, age, years of sports practice, and weekly training volume).

Although sex and pubertal stage are closely related, formal interaction testing between these variables was not performed due to sample stratification and limited statistical power within subgroups. Differences between confidence ellipses were assessed using Hotelling’s T^2^ test, and Mahalanobis distance was used to quantify vector displacement. BIVA 2002^®^ and SpecificBiva^®^ software were used for BIVAc and BIVAsp analyses, respectively. For BIVAc, reference tolerance ellipses were based on an Italian pediatric population,^13^ while for BIVAsp, reference values were derived from athletes with maturational characteristics aligned with peak height velocity.^24^ These reference populations were selected based on their similarity to the age range of the study sample. All analyses were performed using R Studio^®^ (version 2024.04.2+764), adopting a significance level of p≤0.05.

## RESULTS

A total of 78 athletes were evaluated (36 males and 42 females), including soccer (n=2), futsal (n=13), rhythmic gymnastics (n=30), skateboarding (n=3), surfing (n=7), and volleyball (n=23). The mean age of male athletes was 10.8±1.9 years (prepubertal) and 13.9±2.5 years (pubertal). For females, the mean age was 8.1±1.1 years (prepubertal) and 13.0±2.5 years (pubertal). Prepubertal males showed higher R, Z, R/H, Xc/H, and Z/H than pubertal males (p<0.01). Similarly, prepubertal females presented higher R, Xc, Z, R/H, Xc/H, and Z/H than pubertal females (p<0.01). No differences were observed in BIVAsp parameters (Rsp, Xcsp, and Zsp) between maturational groups in either sex (Table 1).

**Table 1.** Descriptive statistics (mean; SD) for the sample characteristics considering male (n=36) and female (n=42) sex for sexual maturation in the prepubertal and pubertal categories.

Female (n=42)	Male (n=36)				Female (n=42)			
	Prepubertal (n=6)	Pubertal (n=30)	p-value	D’	Prepubertal (n=13)	Pubertal (n=29)	p-value	D’
	Mean (SD)	Mean (SD)			Mean (SD)	Mean (SD)		
Body mass (kg)	36.7 (5.7)	56.1	<0.001*	1.13	31.4 (9.5)	50.2 (11.2)	<0.001	1.75
Height (cm)	144.0 (0.1)	164 (0.2)	<0.001^†^	1.26	132 (0.1)	158 (0.1)	<0.001	2.27
Age (years)	10.8 (1.9)	13.9 (2.5)	<0.01^†^	1.28	8.1 (1.1)	13.0 (2.5)	<0.001	2.21
Practice time (years)	6.7 (1.4)	3.1 (2.5)	<0.001*	1.52	2.0 (1.0)	3.6 (2.8)	<0.01	0.68
Weekly training volume (hours)	14.2 (8.6)	9.3 (4.9)	0.233^†^	0.87	8.8 (3.8)	11.9 (4.7)	0.028	0.70
Arm circumference (cm)	21.2 (2.7)	25.7 (4.4)	<0.01^†^	1.05	20.1 (5.0)	24.8 (2.9)	<0.01	1.28
Waist circumference (cm)	61.9 (7.0)	68.6 (8.8)	0.07^†^	0.77	56.4 (13.5)	65.4 (6.4)	0.036	0.99
Calf circumference (cm)	28.5 (2.0)	33.7 (4.3)	<0.001^†^	1.28	25.5 (5.7)	32.0 (2.8)	<0.01	1.66
Resistance (Ω)	677.0 (66.9)	551.0 (100)	<0.01^†^	1.30	761.0 (58.0)	661.0 (69.4)	<0.001	1.51
Reactance (Ω)	82.1 (13.5)	69.9 (9.8)	0.079^†^	1.16	89.6 (15.7)	75.4 (9.7)	<0.01	1.20
Impedance (Ω)	682.0 (67.7)	556.0 (101)	<0.01^†^	1.30	766.0 (59.0)	665.0 (69.5)	<0.001	1.52
R/H (Ω/m)	469.6 (33.7)	344.3 (97.5)	<0.001^†^	1.72	578. (66.5)	420.7 (63.9)	<0.001	2.42
Xc/H (Ω/m)	56.8 (7.4)	43.4 (9.7)	<0.01^†^	1.56	68. (15.5)	47.9 (7.8)	<0.01	1.68
Z/H (Ω/m)	473.0 (33.9)	347.2 (98.0)	<0.001^†^	1.72	582. (67.7)	423.4 (64.1)	<0.001	2.42
Rsp (Ω * cm)	324.7 (51.2)	304.3 (29.2)	0.383*	0.49	342.9 (117.0)	352.0 (53.1)	0.792	0.10
Xcsp (Ω * cm)	39.3 (7.5)	39.0 (5.4)	0.920*	0.05	39.9 (12.3)	40.3 (7.6)	0.909	0.04
Zsp (Ω * cm)	327.1 (51.5)	306.8 (29.3)	0.389*	0.48	345.3 (118.0)	354.4 (53.5)	0.794	0.10

Ω: ohms; m: meter; cm: centimeter; R: resistance; Xc: reactance; Z: impedance; H: height; Rsp: specific resistance; Xcsp: specific reactance; Zsp: specific impedance; SD: standard deviation; D’: Cohen’s d.

Values are presented as mean±SD for normally distributed variables and median for non-normally distributed variables. Normality was assessed using the Shapiro-Wilk test.

*p-value from Mann-Whitney U test; ^†^p-value from Student’s t-test for independent samples.

Source: Prepared by the authors. Study data (2023-2024).

In the overall sample, prepubertal athletes showed higher R, Xc, and Z than pubertal athletes, even after adjustment for sex, age, years of sports practice, and weekly training volume. Height-normalized parameters (BIVAc: R/H, Xc/H, and Z/H) remained higher across all models (Table 2; Table 3). In contrast, no differences were observed for BIVAsp parameters (Rsp, Xcsp, and Zsp) after adjustment (Table 4).

**Table 2.** Comparison of crude bioimpedance vector analysis and maturational status in adolescent athletes.

	Prepubertal (n=19)		Pubertal (n=59)		p-value	R2Adj
	Mean (SD)	95%CI	Mean (SD)	95%CI		
Resistance (Ω)						
Model 1	734.0 (21.9)	690.3-777.7	605.0 (12.4)	580.3-629.8	<0.001	0.246
Model 2	714.9 (18.9)	677.3-752.5	605.9 (10.5)	584.9-626.9	<0.001	0.460
Model 3	655.3 (19.3)	616.9-693.8	626.3 (9.7)	607.1-645.6	<0.001	0.612
Model 4	651.8 (18.6)	614.7-688.9	627.3 (9.3)	608.7-645.8	<0.001	0.641
Model 5	650.1 (18.2)	613.9-686.4	627.6 (9.1)	609.5-645.7	<0.001	0.658
Reactance (Ω)						
Model 1	87.2 (2.6)	82.0-92.4	72.6 (1.5)	69.6-75.5	<0.001	0.226
Model 2	86.1 (2.6)	80.9-91.3	72.6 (1.4)	69.7-75.5	<0.001	0.268
Model 3	83.1 (3.1)	76.9-89.2	73.7 (1.5)	70.6-76.7	<0.001	0.288
Model 4	82.6 (3.0)	76.6-88.7	73.8 (1.5)	70.7-76.8	<0.001	0.313
Model 5	82.5 (3.0)	76.4-88.6	73.8 (1.5)	70.8-76.8	<0.001	0.310
Impedance (Ω)						
Model 1	739.2 (22.0)	695.5-783.0	609.5 (12.5)	584.6-634.3	<0.001	0.248
Model 2	720.2 (19.0)	682.4-757.9	610.3 (10.6)	589.3-631.4	<0.001	0.459
Model 3	660.5 (19.4)	621.8-699.1	630.8 (9.7)	611.4-650.1	<0.001	0.611
Model 4	657.0 (18.7)	619.6-694.3	631.8 (9.4)	613.1-650.4	<0.001	0.639
Model 5	655.3 (18.3)	618.8-691.7	632.1 (9.1)	613.9-650.3	<0.001	0.656

n: sample; SD: standard deviation; Ω: ohms; IC95%: 95% confidence interval; R^2^Adj: adjusted R-squared; Model 1: without covariates (ANOVA); Model 2: adjusted by sex; Model 3: adjusted by sex and age; Model 4: adjusted by sex, age and practice time; Model 5: adjusted by sex, age, practice time and weekly training volume (hours).

Source: Prepared by the authors. Study data (2023-2024).

**Table 3.** Comparison of classic bioimpedance vector analysis and maturational status in adolescent athletes.

	Prepubertal (n=19)		Pubertal (n=59)		p-value	R^2^Adj
	Mean (SD)	95%CI	Mean (SD)	95%CI		
Resistance/Height (Ω/m)						
Model 1	544.3 (20.1)	504.2-584.3	381.9 (11.4)	359.2-404.6	<0.001	0.386
Model 2	528.9 (18.1)	492.8-565.0	382.6 (10.1)	362.4-402.7	<0.001	0.518
Model 3	453.4 (15.4)	422.7-484.1	408.5 (7.7)	393.1-423.9	<0.001	0.760
Model 4	450.4 (14.8)	420.9-480.0	409.3 (7.4)	394.5-424.0	<0.001	0.779
Model 5	448.8 (14.2)	420.4-477.2	409.6 (7.1)	395.4-423.8	<0.001	0.797
Reactance/Height (Ω/m)						
Model 1	64.8 (2.4)	60.0-69.6	45.6 (1.4)	42.9-48.3	<0.001	0.377
Model 2	63.7 (2.4)	59.0-68.4	45.7 (1.3)	43.0-48.3	<0.001	0.421
Model 3	57.2 (2.5)	52.1-62.2	47.9 (1.3)	45.4-50.4	<0.001	0.543
Model 4	56.8 (2.4)	51.8-61.8	48.0 (1.2)	45.5-50.5	<0.001	0.561
Model 5	56.7 (2.5)	51.7-61.6	48.0 (1.2)	45.5-50.5	<0.001	0.566
Impedance/Height (Ω/m)						
Model 1	548.2 (20.2)	507.9-588.4	384.7 (11.5)	361.8-407.5	<0.001	0.387
Model 2	532.8 (18.2)	496.5-569.1	385.5 (10.2)	365.1-405.6	<0.001	0.517
Model 3	456.9 (15.5)	425.9-426.9	411.4 (7.8)	395.9-426.9	<0.001	0.759
Model 4	454.0 (14.9)	424.2-483.8	412.8 (7.5)	397.3-427.1	<0.001	0.778
Model 5	452.3 (14.4)	423.7-480.9	412.5 (7.2)	398.2-426.8	<0.001	0.796

n: sample; SD: standard deviation; Ω: ohms; IC95%: 95% confidence interval; R^2^Adj: adjusted R-squared; Model 1: without covariates (ANOVA); Model 2: adjusted by sex; Model 3: adjusted by sex and age; Model 4: adjusted by sex, age and practice time; Model 5: adjusted by sex, age, practice time and weekly training volume (hours).

Source: Prepared by the authors. Study data (2023-2024).

**Table 4.** Comparison of specific bioimpedance vector analysis and maturational status in adolescent athletes.

	Prepubertal (n=19)		Pubertal (n=59)		p-value	R^2^Adj
	Mean (SD)	95%CI	Mean (SD)	95%CI		
Specific Resistance (Ω *cm)						
Model 1	337.2 (14.8)	307.6-366.7)	327.8 (8.4)	311.0-344.5	0.583	0.001
Model 2	329.6 (14.4)	300.9-358.2	328.1 (8.0)	312.1-344.1	0.565	0.081
Model 3	327.7 (17.5)	292.9-362.5	328.8 (8.7)	311.3-346.2	0.567	0.069
Model 4	326.7 (17.5)	291.7-361.6	329.0 (8.8)	311.6-346.5	0.569	0.064
Model 5	326.1 (17.6)	291.0-361.3	329.2 (8.8)	311.6-346.7	0.570	0.058
Specific Reactance (Ω *cm)						
Model 1	39.7 (1.8)	36.2-43.3	39.7 (1.0)	37.6-41.7	0.973	0.001
Model 2	39.5 (1.8)	35.9-43.1	39.7 (1.0)	37.6-41.7	0.973	0.001
Model 3	41.7 (2.2)	37.4-46.0	38.9 (1.1)	36.8-41.1	0.972	0.009
Model 4	41.6 (2.2)	37.2-45.9	38.9 (1.1)	36.8-41.1	0.972	0.001
Model 5	41.6 (2.2)	37.2-46.0	38.9 (1.1)	36.7-41.1	0.973	0.001
Specific Impedance (Ω *cm)						
Model 1	339.5 (14.9)	309.9-369.2	330.2 (8.4)	313.9-369.2	0.587	0.001
Model 2	332.0 (14.5)	303.1-360.8	330.6 (8.1)	314.5-346.6	0.570	0.079
Model 3	330.4 (17.5)	295.4-365.3	331.1 (8.8)	313.6-348.6	0.572	0.067
Model 4	329.3 (17.6)	294.2-364.5	331.4 (8.8)	313.8-349.0	0.573	0.062
Model 5	328.8 (17.7)	293.5-364.2	331.5 (8.8)	313.8-349.1	0.575	0.055

n: sample; SD: standard deviation; Ω: ohms; IC95%: 95% confidence interval; R^2^Adj: adjusted R-squared; Model 1: without covariates (ANOVA); Model 2: adjusted by sex; Model 3: adjusted by sex and age; Model 4: adjusted by sex, age and practice time; Model 5: adjusted by sex, age, practice time and weekly training volume (hours).

Source: Prepared by the authors. Study data (2023-2024).

**Figure 1.**
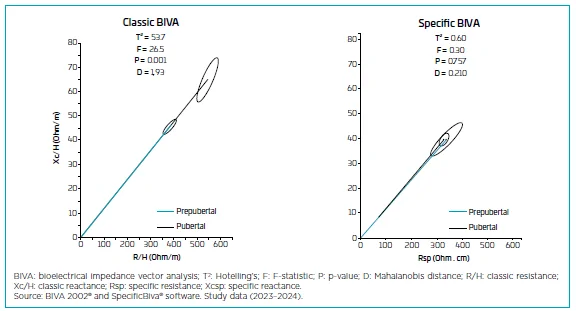
Mean impedance vectors with 95% confidence ellipses of classic and specific bioelectrical impedance vector analysis of prebubertal and pubertal adolescent athletes.

BIVAc parameters showed increasing explained variance across models. Adjusted R^2^ for R and Z increased from ~0.24 (Model 1) to ~0.66 (Model 5), while height-adjusted parameters reached ~0.80 in fully adjusted models (Table 2; Table 3). BIVAsp parameters showed minimal explained variance (adjusted R²≈0.00-0.08) across all models (Table 4). BIVAc showed distinct separation between prepubertal and pubertal groups, whereas BIVAsp ellipses overlapped. Hotelling’s T^2^ confirmed differences for BIVAc (p=0.001), but not for BIVAsp (p=0.757) (Figure 1).

**Figure 2.**
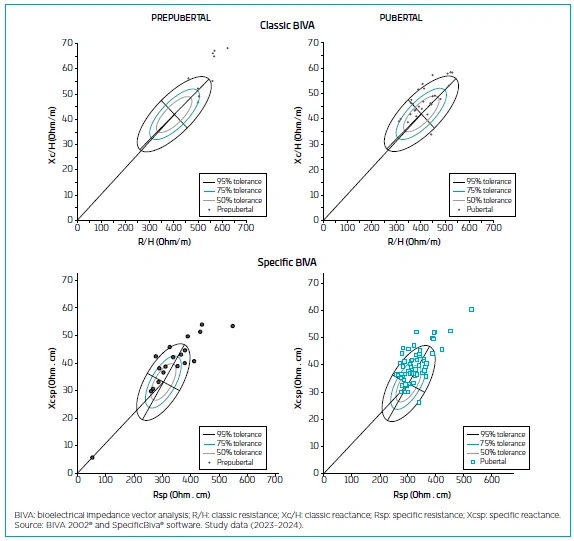
Impedance vectors in the 50, 75, and 95% tolerance ellipses of the classic and Specific bioelectrical impedance vector analysis for Prepubertal and Pubertal adolescent athletes.

In Figure 2, BIVAc vectors were predominantly located above the center and distributed between the right and left quadrants. Prepubertal athletes were more frequently positioned between the 75 and 95% tolerance ellipses or outside them, whereas pubertal athletes were mainly distributed within the 50, 75, and 95% ellipses. In contrast, BIVAsp vectors showed similar distribution patterns between prepubertal and pubertal groups across the tolerance ellipses of the reference population.

## DISCUSSION

The main findings showed that prepubertal athletes had higher R, Xc, and Z than pubertal athletes, even after adjustment for sex, age, training experience, and weekly training volume. These differences remained when parameters were normalized by height (BIVAc) but were no longer observed when normalized by body circumferences and height (BIVAsp). These discrepancies likely reflect differences in normalization strategies. BIVAc adjusts parameters only by height, reducing the effects of conductor length but remaining influenced by body dimensions.^12^ ^,^ ^13^ In contrast, BIVAsp incorporates arm, waist, and calf circumferences to account for cross-sectional areas and body geometry, improving tissue-specific interpretation.^8^ ^,^ ^9^ ^,^ ^24^ ^,^ ^25^ ^,^ ^26^ ^,^ ^27^

Beyond statistical significance, the magnitude of these differences is supported by the adjusted R^2^ values. BIVAc parameters showed increasing explained variance across adjusted models, particularly for height-normalized variables, indicating a strong influence of maturation and body size on impedance patterns.^12^ ^,^ ^13^ In fully adjusted models, R^2^ values reached high levels (~0.80), whereas BIVAsp parameters remained consistently low (~0.00-0.08), indicating that pubertal stage explained minimal variance after accounting for body geometry.^8^ ^,^ ^9^ ^,^ ^27^ This indicates that part of the vector displacement observed in BIVAc reflects growth-related changes rather than tissue properties alone.^12^ ^,^ ^24^

Higher raw and height-normalized bioelectrical values in prepubertal athletes (R, Xc, and Z; R/H, Xc/H, and Z/H) were accompanied by distinct vector displacement patterns compared to pubertal athletes, even after adjustments. Although age and maturation are biologically related and may introduce potential multicollinearity, they capture distinct aspects of growth. Age was included as a covariate to account for chronological variation, and differences between groups remained significant after adjustment, reinforcing that maturational status reflects biological development beyond chronological age.^21^ ^,^ ^24^ Thus, these variables, although correlated, capture different aspects of growth in young athletes.

In prepubertal athletes, BIVAc vectors were in the upper quadrants, indicating leaner profiles and higher hydration.^7^ ^,^ ^12^ These vectors were also positioned closer to the 75-95% tolerance ellipses or beyond them, reinforcing a profile of lower fluid volume and higher resistance. In pubertal athletes, vectors were distributed toward the upper- and lower-left quadrants, suggesting a trend toward both athletic and overweight/obese profiles and more frequently located within the central tolerance ellipses (50-75%), indicating greater fluid content and body mass.^7^ ^,^ ^12^ Puberty is characterized by increases in lean mass, fat mass, and body water, with sex-specific differences in fat and fat-free mass.^25^ ^,^ ^26^ Linear growth, particularly height, plays a key role in these changes and helps explain differences in R/H and Xc/H between maturational stages.^21^

Regarding BIVAsp, no differences were observed between prepubertal and pubertal athletes, suggesting that accounting for body geometry attenuates maturational effects. This approach emphasizes tissue properties rather than conductor length, thereby better representing conductive and resistive tissues.^8^ ^,^ ^9^ ^,^ ^27^ BIVAsp may also homogenize vector displacement, as circumferences vary proportionally less than height during puberty.^24^ ^,^ ^25^ These findings align with previous studies in youth athletes showing limited influence of maturity on BIVAsp parameters.^24^

Waist circumference was higher in pubertal females but did not differ in males. This sex-specific difference reinforces known patterns of fat distribution during puberty, particularly the greater accumulation of adipose tissue in females. As an indicator of fat distribution, particularly visceral fat, it may influence bioelectrical properties.^28^ ^,^ ^29^ However, it did not explain maturational differences in this sample, as BIVAsp normalization attenuated group differences.^30^ Thus, BIVAc appears more sensitive to growth-related changes, whereas BIVAsp may neutralize part of this variation.

The choice between BIVAc and BIVAsp should be guided by study objectives and population characteristics. BIVAc was more strongly associated with growth and maturation, whereas BIVAsp emphasized body geometry and attenuated maturational differences.

Due to the cross-sectional design, causal relationships and individual maturational trajectories cannot be established, and reverse causality cannot be excluded. However, the study still identifies relevant associations between maturation and bioelectrical impedance patterns. The relatively small sample size may limit precision and generalizability, particularly in subgroup analyses, despite the use of post hoc analysis. Italian reference populations were used due to the lack of Brazilian data, which may introduce bias related to ethnic and anthropometric differences. Additionally, grouping pubertal and post-pubertal participants may have reduced sensitivity to later maturational contrasts, and self-assessed sexual maturation, although validated, may allow some misclassification.

Although standardized procedures and relevant covariates were included, heterogeneity in maturational stages and the absence of more complex modeling to address confounding, collinearity, and interaction effects should be considered. The predominance of athletes from artistic and rhythmic gymnastics may also limit generalizability to other sports.

Despite these limitations, this study has important strengths. It provides original data on child and adolescent athletes analyzed by maturational status using raw parameters, BIVAc, and BIVAsp within the same sample, enabling direct comparison between approaches. The consistency of findings supports robustness, even with a relatively small sample, and improves understanding of how BIVA strategies behave during growth and maturation.

BIVAsp did not distinguish prepubertal from pubertal athletes, whereas BIVAc was more strongly associated with maturational status. These findings reinforce that normalization strategies influence how bioelectrical parameters relate to growth and maturation. Future longitudinal studies in diverse populations are needed to clarify how BIVAc and BIVAsp reflect maturational and body composition changes.

In conclusion, prepubertal athletes showed higher R, Xc, and Z than pubertal athletes in both raw and height-normalized analyses (BIVAc), whereas these differences disappeared when parameters were normalized by body circumferences and height (BIVAsp). These findings highlight the importance of the normalization strategy when examining bioelectrical parameters across maturational stages. Rather than indicating greater sensitivity, the results suggest that BIVAc and BIVAsp are differently associated with pubertal status. Thus, this study contributes to understanding how normalization approaches influence the interpretation of BIVA during growth and maturation.

## Data Availability

The database that originated the article is available with the corresponding author.
